# Electrified Interactions of Polyzwitterions with Charged Surfaces: Role of Dipole Orientation and Surface Potentials

**DOI:** 10.1021/acs.langmuir.4c00343

**Published:** 2024-03-27

**Authors:** Chia-Hsuan Lin, Jhih-Guang Wu, Hsun-Hao Lin, Shyh-Chyang Luo

**Affiliations:** † Department of Materials Science and Engineering, National Taiwan University, No. 1, Sec. 4, Roosevelt Road, Taipei 10617, Taiwan; ‡ Institute of Biomedical Engineering and Nanomedicine, National Health Research Institutes (NHRI), Miaoli County 35053, Taiwan

## Abstract

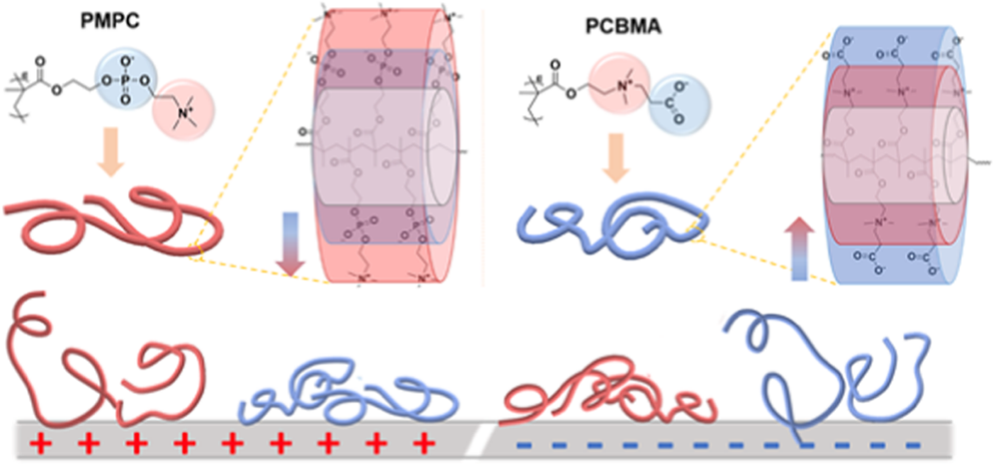

The zwitterionic groups possess strong dipole moments, leading to inter- or intrachain interactions among zwitterionic polymers. This study aims to demonstrate the interaction of polyzwitterions poly(2-methacryloyloxyethyl phosphorylcholine) (PMPC), and poly(carboxybetaine methacrylate) (PCBMA) with electrified surfaces, despite their electrically neutral nature. We studied the adsorption of polyzwitterions and their monomers on electrified surfaces by using an electrochemical quartz crystal microbalance with dissipation (EQCM-D). The interaction between zwitterionic molecules and charged surfaces is explored by adjusting the surface potentials. Interestingly, the adsorption of polyzwitterions can be influenced by external potential, primarily due to the formation of polyzwitterions restricting the mobility of zwitterionic groups, affecting the adsorption behavior of polyzwitterions based on the surface potential. The impact is determined by the arrangement of positive and negative ions within the zwitterionic groups, which are the dipole orientation. Additionally, surface potentials determine the adsorption rate, amount, and chain conformation of the adsorbed thin polyzwitterion layers. The effect of ionic strength was investigated by introducing electrolytes into the aqueous solutions to assess the range of influenced surface potentials.

## Introduction

Zwitterionic molecules, which contain functional groups with equal positive and negative charges, are highly promising polymers for antifouling purposes. Their ease of structural modification and the resistance to unwanted adsorption enhance the sensitivity and specificity of bioanalysis under physiological conditions.^1−4^ These molecules are numerously found in biological systems and are often used as building blocks for synthetic polyzwitterions. Phosphorylcholine (PC), sulfobetaine (SB), and carboxybetaine (CB) are the most utilized zwitterionic moieties, which all contain a quaternary ammonium cation but different anions, namely, phosphate anions, sulfite, and carboxylate, respectively. In an aqueous environment, the opposite charges in zwitterionic functional groups generate massive local dipole moments, resulting in strong dipole–dipole and charge–dipole interactions.^5^ Accordingly, hydration layers can be formed through these strong interactions among zwitterion moieties and water as long as the polyzwitterions are well arranged to create a stable interface. These interactions are believed to be the origin of the powerful antifouling properties of polyzwitterions, making them highly applicable in various practical settings.^6−9^

Moreover, the formally charged but net-neutral structure gives polyzwitterions numerous unique features. One of these features is the antipolyelectrolyte effect,^10^ which is a prominent effect of polyzwitterion in response to the interaction with added ions. For general polyelectrolytes with a net positive or negative charge, their polymer chains associate in solutions when salts are present, resulting in a collapsed conformation. However, polyzwitterions exhibit chain conformation expansion. Due to their electrostatic dipole–dipole interactions, polyzwitterion chains adopt a collapsed conformation in salt-free or low-ionic-strength aqueous environments. Conversely, the chains start to extend with increasing ionic strength due to the screening of inter/intrachain associations weakened by the free ions in the solutions.^11−13^

In the past few decades, the behavior of polyelectrolyte adsorption has been widely studied. Generally, the attracted polyelectrolyte on an oppositely charged surface would deposit and form a steady, thin layer, providing a rapid, facile, and versatile method for surface modification. Through their versatility, the deposition of polyelectrolytes is found in many applications.^14,15^ In recent years, there has been increasing research interest in exploring the responsiveness of polyelectrolytes to electrical fields and examining their structural behavior on electrified substrates.^16,17^ While polyelectrolyte adsorption behavior has been widely investigated, polyzwitterion adsorption has yet to be well studied. Previous studies mainly focused on polyampholyte adsorption,^18−21^ where its structure carries both basic and acid groups along the molecular backbone chain. The molecular design of net-neutral polyampholytes could range from block through to totally alternating monomer sequences. Khan et al.^22^ investigated the effect of charge sequences of weakly coupled polyampholytes, whose adsorption is induced by the polarization of the chain by the charged surface. They used simulations to analyze the adsorption behavior on negatively charged surfaces. For neutral diblock polyampholytes, adsorption of the positively charged side was promoted as expected, and the negatively charged half dangled out from the surface. Conversely, the alternating chain sequences exhibited minimal adsorption, since this chain was hardly polarized and preferred the bulk over the charged surface. As for polyzwitterions, such as polybetaine moieties, they illustrate properties different from those of the polyampholytes mentioned above. Their zwitterionic moieties are on the same monomeric unit, remaining electrically neutral without molecular-level segregation. Hence, a study for resolving the interaction between the charged surface and zwitterionic molecules, including zwitterionic monomers and polyzwitterions, is highly desirable.

Herein, we address this problem using electrochemical quartz crystal microbalance with dissipation (EQCM-D) at different surface potentials on indium tin oxide (ITO) electrodes to illustrate how electrically neutral polyzwitterions interact with charged surfaces. This study investigated the adsorption behavior of two polyzwitterions: poly(2-methacryloyloxyethyl phosphorylcholine) (PMPC) and poly(carboxybetaine methacrylate) (PCBMA). PMPC carries opposite dipole moment directions to PCBMA. The passive electrodeposition of these polymers was conducted on the ITO surface using EQCM-D by applying negative to positive bias on the electrodes. In addition to polyzwitterions, the adsorption behavior of their individual monomers was also investigated to elucidate the difference in the capability of the dipole moment rotating. The effect of ionic strength on these zwitterionic molecules was further discussed in the presence of lithium perchlorate (LiClO_4_) as an electrolyte.

## Materials and Methods

### Materials

MPC, ethyl α-bromoisobutyrate (EBIB), 2,2′-bipyridine (Bpy), and 2,2,2-trifluoroethanol (TFE) were purchased from Sigma-Aldrich. CBMA was purchased from Tokyo Chemical Industry. CuBr and LiClO_4_ were purchased from Alfa Aesar. Methanol was purchased from Aencore Chemical. Deionized (DI) water was prepared using a MICRA system (MICR00001423, Taiwan) to achieve a resistivity of 15 MΩ·cm.

### Preparation of Polyzwitterions

To prepare two kinds of zwitterionic polymers, PMPC and PCBMA, we performed atom transfer radical polymerization (ATRP) under the respective fabrication conditions to obtain optimal results. Table S1 lists the ratio of monomer to free initiator (EBIB) of PMPC and PCBMA. MPC monomers were first agitated in ether to eliminate the inhibitor and centrifuged for 3 min (7000 rpm). After centrifugation, the centrifuge tube was evacuated for 30 min. The purified MPC monomers were next dissolved in 8.5 mL of methanol into a glass tube with EBIB and purged with N_2_ gas for 15 min. Bpy and CuBr were added into a flask with a stir bar and subjected to evacuation and filling with N_2_ gas three times. Afterward, the dissolved MPC solution was transferred into the flask by a syringe to start the ATRP. The reaction was carried out for 24 h at room temperature.

CBMA monomers were dissolved in 5 mL of methanol into a glass tube with EBIB and purged with N_2_ gas for 15 min. Bpy and CuBr were added into a flask with a stir bar and subjected to evacuating and filling with N_2_ gas three times. Afterward, the dissolved CBMA solution was transferred into the flask by a syringe to start the ATRP. The reaction was carried out for 1 h at room temperature.

### Gel Permeation Chromatography (GPC) Measurements

We used GPC to determine the molecular weights of polymers with a 1525 HPLC pump and a Waters 2414 RI detector. This system has two Waters columns (Ultrahydrogel columns, WAT-011520, and WAT-011530). Polymers were prepared by dissolving them in double-distilled water containing 0.2 M sodium nitrate. A 10% methanol aqueous solution was used as an eluent, and poly(ethylene glycol) (PEG) with five different molecular weights was used as the standard polymer for calibration.

### Fourier Transform Infrared Measurements

The functional groups on polyzwitterions were characterized by attenuated total reflection Fourier transform infrared (ATR-FTIR) spectroscopy in the range of 2000–750 cm^–1^ at a resolution of 4.0 cm^–1^ with 64 scans.

### QCM-D Measurements

The zwitterionic molecules’ adsorption/desorption was conducted using a QCM-D (QSense Explorer and Analyzer system) with the quartz crystal resonator (QSX 301 Au sensor or QSX900 ITO sensor) set at a 5 MHz fundamental resonant frequency. The measurements were undertaken in the QFM401 QSense Flow module, and a flow rate of 36.5 μL/min was controlled by using a tubing pump. All QCM-D measurements were repeated at least three times to ensure the accuracy and reproducibility of the data. The data recorded the changes in energy dissipation factor (Δ*D*) and normalized frequencies (Δ*f_n_*/*n*) as a function of time. In this study, all of the frequency and dissipation measurements were taken from the 3rd overtone (*n* = 3) because the 1st overtone is so sensitive that any vibration may interfere with the balance, and the 5th to the 13th could provide almost comparable information. Hence, Δ*f* is used to represent Δ*f*_3_/3 for simplicity. In the case of soft coatings such as polymers in a liquid phase, the viscous environment dissipates the oscillation energy. The change in the frequency values obtained from the QCM-D test is positively correlated with the mass of adsorption, and the amount of adsorption can be estimated only by identifying the oscillation frequency drop. Moreover, the concept of energy dissipation (*D*) is introduced to the QCM-D system to extend the application of the QCM measurements. The energy dissipation of the QCM-D resonator was defined as *D* = *E*_d_/(2π*E*_s_), where *E*_d_ is the loss modulus and *E*_s_ is the storage modulus. Higher dissipation indicates faster energy decay at the oscillation of sensors, and it usually presents the adsorption of large or soft molecules on the quartz. On the other hand, the lower energy dissipation is generally followed by the release of soft or hydrated molecules.

Before the QCM test was started, we confirmed that a stable baseline had been reached in running solutions. The frequency and energy dissipation changes can be extracted by subtracting the equilibrium baseline value corresponding to background solutions. All of the QCM measurements were performed at 25 °C.

### EQCM-D Measurements

In addition to QCM-D measurements, we monitored the same phenomena under potentiostatic bias conditions. The electrically assisted experiments were undertaken in the QEM 401 QSense electrochemical module integrated with a potentiostat to apply an external surface potential. A three-electrode setup was employed by introducing a Ag/AgCl leak-free reference electrode with 3.4 M KCl. The EQCM-D measurements reached an equilibrium value initially under a constant flow of solutions that persisted for a few minutes before the experiments began.

### *D*–*f* Plots and the Value of |Δ*D*/Δ*f*|

The shift in dissipation versus frequency plots (*D*–*f* plots) is commonly used to investigate the viscoelastic properties of the molecular conformation at the interfaces. In other words, the slope changes in *D*–*f* plots (values of |Δ*D*/Δ*f*|) can allow the dynamic changes in the conformation of soft materials. This methodology has been used to compare the adsorption of polymers, proteins, polysaccharides, and cell adhesion on different surfaces under different conditions. When the adsorbed layer leads to a large *D* in a unit mass (frequency), namely, a higher |Δ*D*/Δ*f*|, the viscoelastic properties of this layer are considered loose conformation or hydrated layer. On the other hand, for *a* lower |Δ*D*/Δ*f*|, the adsorbed layer is considered dense, collapsed conformation or less hydrated layer.

## Results and Discussion

To investigate the adsorption behavior of polyzwitterions with opposite dipole moment directions, we prepared PMPC and PCBMA as candidates. ATRP fabricated the polymers; their molecular weights were 1.3 × 10^4^ and 9.1 × 10^3^ g/mol with similar repeat units. More detailed information is summarized in Table S1 and Figure S1. As for the characterization of their functional groups, the ATR-FTIR spectra of PMPC and PCBMA are illustrated in Figure S2. The results all suggested that the polyzwitterions had been successfully synthesized through ATRP reactions, which possess well-controlled molecular weight with a relatively small polydispersity index compared to other radical polymerizations. It helps us further elucidate the conformation change of polymer chains on these two polyzwitterions.

**Figure 1:**
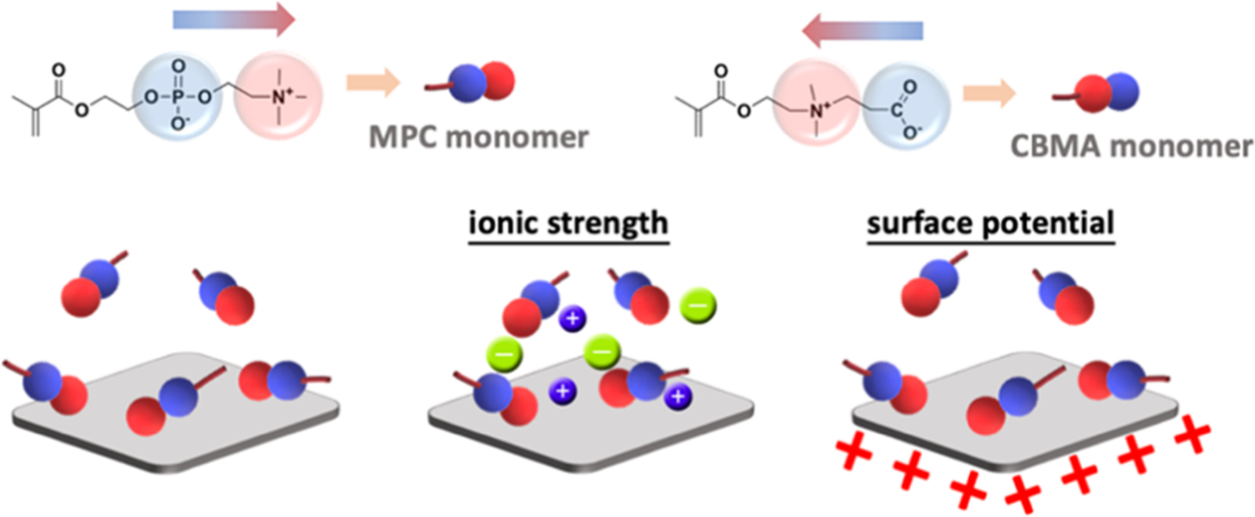
Chemical structure and schematic dipole of MPC and CBMA monomers and the scope of this study.

To better illustrate the different influence of surface potential on the adsorption of polyzwitterions compared to zwitterionic molecules, we initially investigated the adsorption behavior of zwitterionic monomers, such as MPC and CBMA, using QCM-D as shown in Figure 1. By applying an external surface potential, we evaluated the adsorption behaviors of these zwitterionic monomers and polymers on an electrified surface. In QCM experiments, the most commonly used sensor is the Au sensor. We have previously used EQCM-D to assess the antifouling properties of conducting polymer-coated Au sensors.^23−25^ However, when the bare Au sensors were used for EQCM-D, the results exhibited some unexpected shifts or intense noises in the background of the baseline under high applied potentials (Figure S3). We suggested that these unstable signals were attributed to the formation of tiny hydrogen bubbles at the Au surfaces. Therefore, we replaced the original Au sensors with the ITO-coated sensors to avoid the measurements from the interference of bubble formations,^26^ and decided to investigate the effect of surface potential on the adsorption behaviors of zwitterionic monomers and polymers on the ITO-coated sensors. The ITO-coated sensors have higher overpotentials and wider electrochemical windows than Au sensors, providing stable QCM results, even when surface potentials are applied.

**Figure 2:**
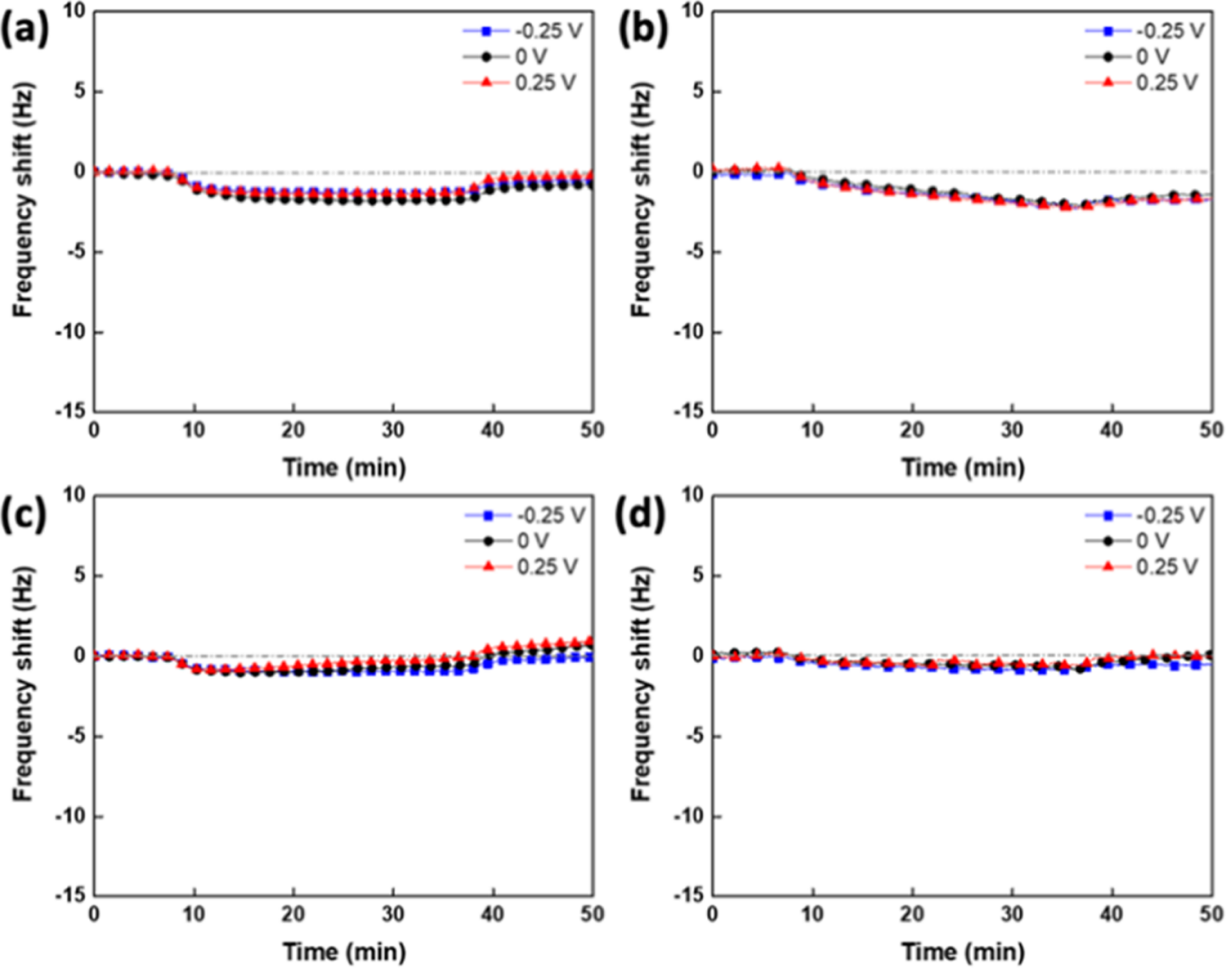
Real-time Δ*f* during the adsorption process of (a) MPC in DI water, (b) CBMA in DI water, (c) MPC with 100 mM LiClO_4_, and (d) CBMA with 100 mM LiClO_4_ under external potentials of −0.25, 0, and 0.25 V.

It is noted that the zwitterionic moieties in polyzwitterions are fixed to their main chains, which means that their dipole moments cannot freely rotate and change directions. The different adsorption behaviors of monomers than polymers are expected because the individual zwitterionic monomers, without bonding to the main chains, could freely move in the aqueous environment. To evaluate the surface potential effect, we conduct the EQCM-D measurements by applying an external potential on the sensors’ surface. We set −0.25 V as a negative and 0.25 V as a positive surface potential compared to 0 V vs Ag/AgCl in aqueous environments, as shown in Figure 2. The concentration of the two zwitterionic monomers is 1 mg/mL, and the ionic strength effect was further discussed by adding 100 mM LiClO_4_ in DI water. The choice of LiClO_4_ to modulate the ionic strength in this study is due to the electrochemical stability of ClO_4_^–^ ions. Cl^–^ ions may generate gas upon applying oxidative potential, which affects the EQCM-D measurements.^27^

MPC adsorption in DI water caused only a slight frequency drop (Δ*f* < 1.5 Hz) and shifted back to the initial frequency baseline after changing the mobile phase to DI water, as shown in Figure 2a. This result suggests that MPC monomers are barely bound to the ITO surface. Moreover, no distinct changes were observed when different surface potentials were applied to the ITO surfaces. The adsorption of CBMA monomers using different surface potentials on the ITO surface exhibited similar behaviors to the MPC monomers, where the Δ*f* values were less than 2 Hz in DI water. The adsorption of CBMA on the ITO surface might be due to the interaction between the carboxyl groups and the ITO surfaces. The adsorption amount displayed independence from the external surface potential. We also raised the concentration of zwitterions to 10 mg/mL in DI water, as shown in Figure S4. Still, MPC and CBMA adsorption barely depended on the external surface potential.

**Figure 3:**
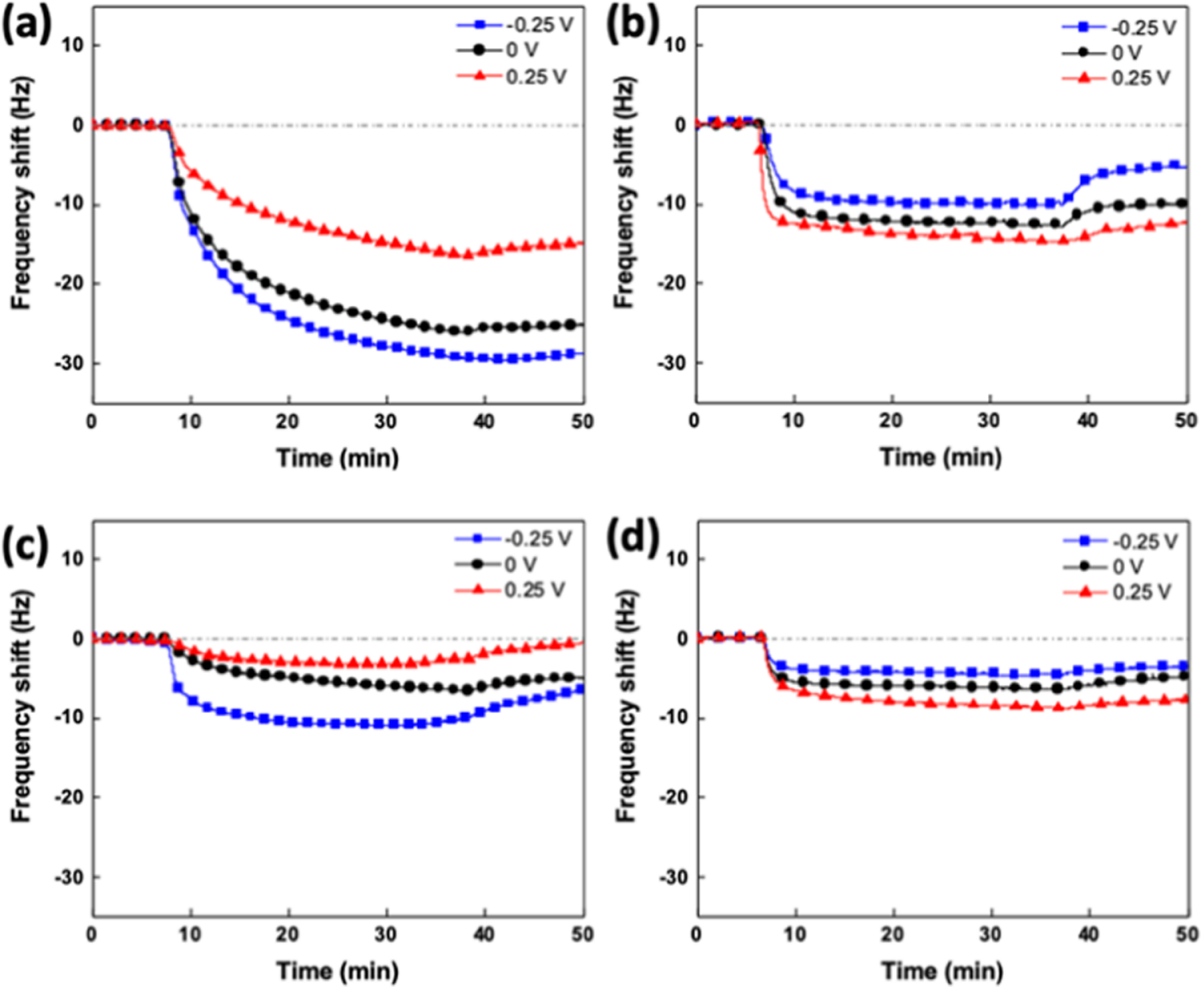
Real-time Δ*f* during the adsorption process of (a) PMPC and (b) PCBMA polymers in DI water and (c) PMPC and (d) PCBMA in 100 mM LiClO_4_ on the ITO surface with an external potential at −0.25, 0, and 0.25 V.

Next, we explored the adsorption of polyzwitterions on the ITO-coated sensors using the same setup, including the PMPC and PCBMA polymers in DI water and LiClO_4_, as shown in Figure 3. Compared to monomers, the zwitterionic polymer presented more adsorption on ITO-coated sensors. Furthermore, the adsorption was influenced by the applied potentials. As shown in Figure 3a, we examined the adsorption behavior of PMPC polymers dissolved in DI water with external surface potentials of −0.25, 0, and 0.25 V. Different initial rates of PMPC adsorption were observed, where 0.25 V presented a slower rate and −0.25 V showed a faster one. This result indicates that externally applied potential on the surfaces affects the kinetic adsorption of polyzwitterions. The remaining adsorbed PMPC decreased as the applied potential increased from −0.25 to 0.25 V after the mobile phase changed to pure DI water. Compared to the Δ*f* value at 0 V, the adsorbed PMPC increased on a negatively charged surface and decreased on a positively charged one, exhibiting the consistency of the effect of surface potential on the initial adsorption rates. On the other hand, as shown in Figure 3b, the adsorption results of PCBMA presented the opposite trend regarding the applied potentials compared to the adsorption of PMPC. The remaining adsorbed PCBMA increased as the applied potential increased from −0.25 to 0.25 V in pure DI water. According to the results of adsorption behaviors on the electrified ITO surfaces, PMPC and PCBMA exhibited an opposite tendency to the applied surface potentials. We proposed that the different interactions between polyzwitterions and surface potentials originated from the opposite direction of cations and anions, the dipoles, on PC and CB moieties. Although zwitterionic moieties are electrically neutral, they still present inter- and/or intrachain interactions due to the dipole moments. We suggest that an external electrical field might affect the dipole moments in zwitterionic moieties. PMPC and PCBMA polymers could be attracted or repulsed following the interaction between the surface potentials and the dipole moment direction of zwitterionic moieties bonded to the main chains of polymers. In comparison to the adsorption of PCBMA, PMPC typically exhibits a higher adsorption for ITO surfaces. Based on earlier studies, phosphonic acid groups have demonstrated a strong tendency to adhere to hydroxylated ITO surfaces.^28^ We speculate that this could account for the higher adsorption of PMPC compared to PCBMA. In addition to the quantity of adsorption being influenced by the electrified potential, we can also infer the rate of adsorption from the rate of frequency decrease, which corresponds to the slope. The data indicate that when changing the surface potential, not only does the quantity of adsorption change but also the rate of adsorption varies due to changes in the dipole orientation. Specifically, at +0.25 V, the rate of PMPC adsorption decreases while promoting the rate of PCBMA adsorption.

The increasing ionic strength in aqueous solutions would influence the adsorption of molecules carrying charges to some extent, such as polyelectrolyte adsorption. The ions dissolved in aqueous solutions might accumulate in different orders at the interface between the liquid and solid phases of electrified surfaces. The distribution of the ionic layer is called the electrical double layer, and the extended distance of the diffuse layer of the electric double layer in the solutions is λ_D_, which can also be viewed as the thickness of the electric double layer.^29,30^ The influence of external surface potential would be weakened with increasing ionic strength.^31,32^ On the other hand, the ionic strength would have different impacts on the polyzwitterions’ conformations and properties. To clarify the synergistic effects of the ionic strength and surface potential, we repeated the EQCM-D measurement, replacing the DI water with 100 mM LiClO_4_ aqueous solutions. As shown in Figure 3c, the adsorption behavior of PMPC on electrified ITO surfaces in 100 mM LiClO_4_ solutions still followed the trend of interaction between the surface potentials and PMPC chains. However, compared to the results of PMPC adsorption at the same surface potentials but in DI water, all were reduced in the presence of 100 mM LiClO_4_. Since it is known that the conformation of PMPC exhibits barely dependency on the ion concentration in the aqueous environment, we suggest the reduction of PMPC adsorption was attributed to the shielding of the electric double layer via the added salts. Following the same procedure, the surface potential effect on the adsorbed amount of PCBMA was still observed. The results were consistent with the tendency of the adsorption behavior in DI water. Similar to the PMPC adsorption, all reduced in the presence of 100 mM LiClO_4_ due to the weakened influence of external surface potential. As the extended distance of the diffuse layer of the electric double layer decreased, the extent of polyzwitterion adsorption was also extenuated in the LiClO_4_ solution.

**Figure 4:**
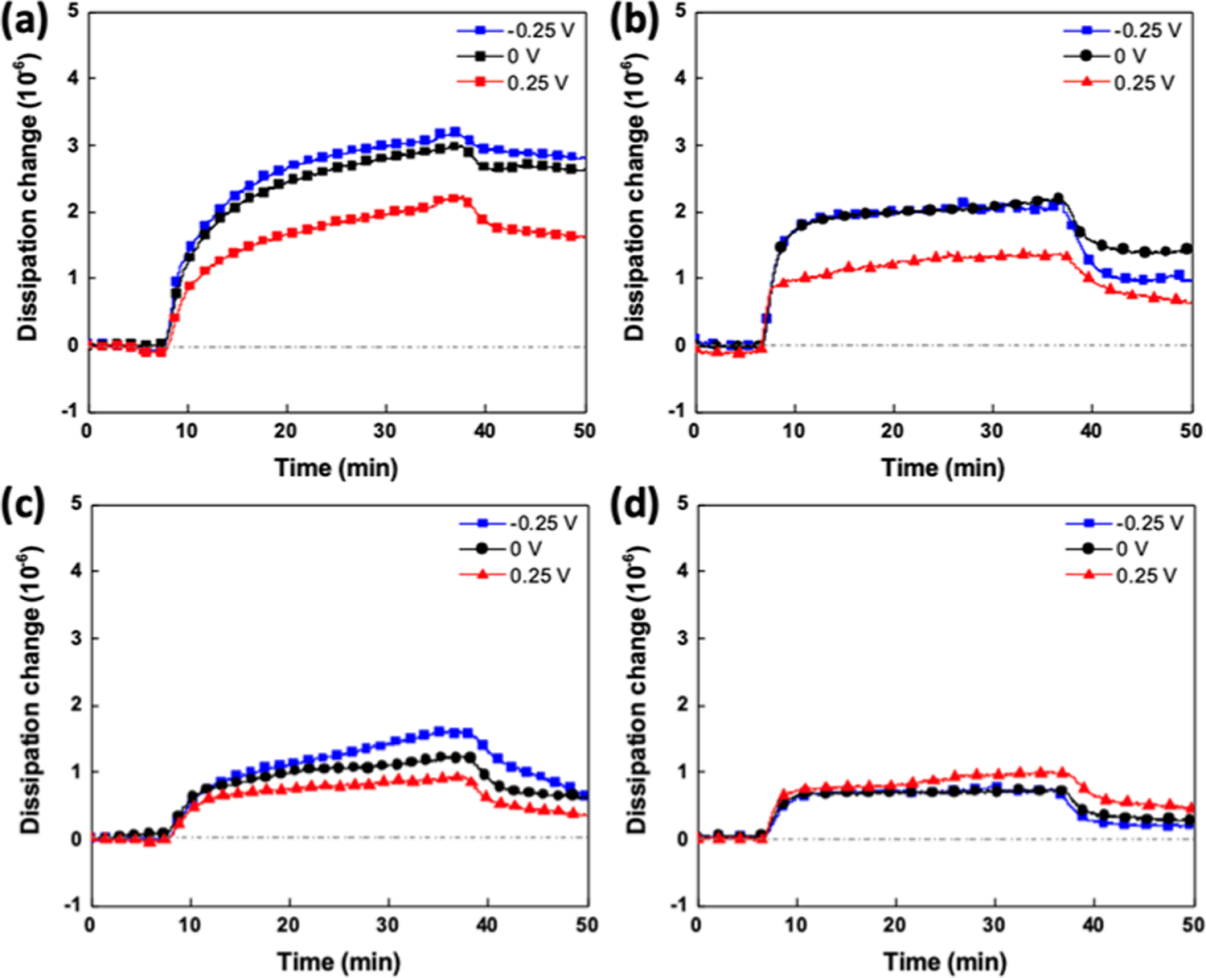
Real-time Δ*D* during the adsorption process of (a) PMPC and (b) PCBMA on ITO-coated sensor in DI water and (c) PMPC and (d) PCBMA in 100 mM LiClO_4_ on the ITO surface with an external potential at −0.25, 0, and 0.25 V.

To better analyze the adsorption behaviors of polyzwitterions, we not only monitored the real-time frequency shift and energy dissipation change (Δ*f* and Δ*D*) during the EQCM-D measurements but also evaluated the viscoelasticity of the adsorbed polymer layers by extracting and calculating the values of |Δ*D*/Δ*f*|. The slope changes in *D*–*f* plots (values of |Δ*D*/Δ*f*|) are commonly utilized to elucidate the viscoelastic properties of the soft, adsorbed thin layers.^33−35^ Generally, a higher |Δ*D*/Δ*f*| is considered the loose conformation or hydrated status of the adsorbed layer. By contrast, for a lower |Δ*D*/Δ*f*|, the adsorbed layer is considered dense, collapsed conformation, or less hydrated layer. Regarding the assessment of protein or polymer adsorption using QCM-D, since the adsorption process of these macromolecules is somewhat random, with molecules stacking in different orientations, there can be variations in the quantity (Δ*f*) of the polymer layer measured each time. Therefore, there may be some deviation. As a result, the Δ*D*/Δ*f* value is more akin to normalizing the impact of quantity and can be used more accurately as an indicator of stiffness. The real-time Δ*D* during the adsorption process of PMPC and PCBMA polymers in DI water and in 100 mM LiClO_4_ on the ITO surface with an external potential at −0.25, 0, and 0.25 V are shown in Figure 4. The increasing energy dissipation on the surface is mainly due to the attachment of highly swelled and hydrated polymers on the ITO surface. The values of |Δ*D*/Δ*f*| at different surface potentials were further compared to evaluate the conformations of polymers adsorbed on the ITO surface and are summarized in Table 1.

**Table 1:** Average Values of |Δ*D*/Δ*f*| for PMPC and PCBMA Adsorbed on the ITO Surface in DI Water and LiClO_4_ with an External Potential at −0.25, 0, and 0.25 V

	DI water		with LiClO_4_	
|Δ*D*/Δ*f*| (V)	PMPC	PCBMA	PMPC	PCBMA
–0.25	0.0972	0.1843	0.0984	0.0632
0	0.1048	0.1430	0.1252	0.0634
0.25	0.1108	0.0516	0.1422	0.0635

**Figure 5:**
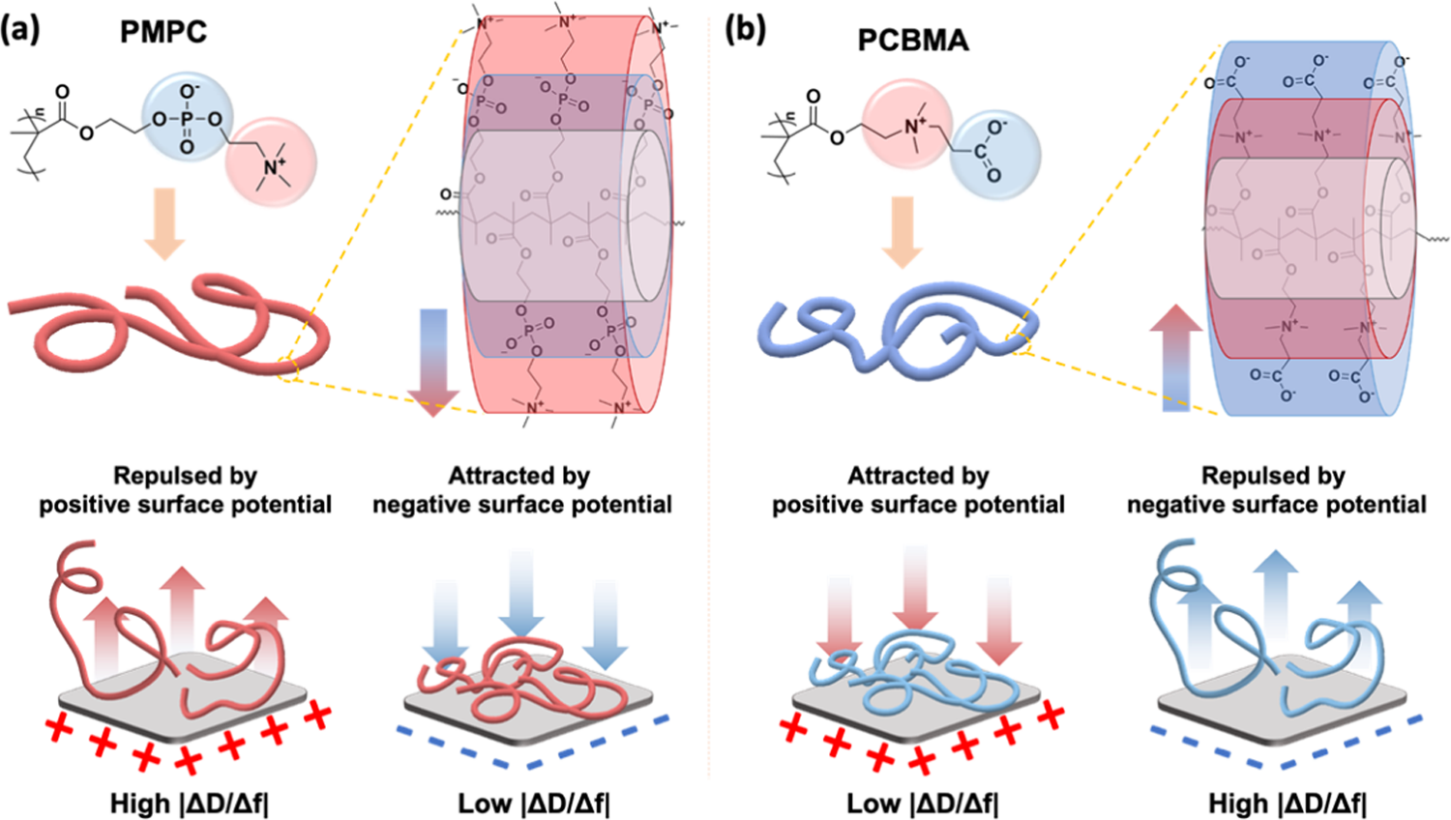
Schematic mechanism of the surface potential effect on (a) PMPC and (b) PCBMA adsorption, including an illustration of a polyzwitterion chain and the surface potential effect on the viscoelasticity change of adsorbed layers at a positive or negative surface potential.

Regarding PMPC adsorption, the |Δ*D*/Δ*f*| slightly increased from −0.25 to 0.25 V. The results suggested that the adsorbed PMPC thin layer on a negatively charged surface formed a denser conformation than the layer on a positively charged one. Based on the EQCM-D readouts, we infer that a negative external surface potential caused PMPC to adsorb tightly, forming *a* dense thin layer with more adsorbed PMPC on the ITO surface. The |Δ*D*/Δ*f*| for PCBMA adsorption exhibited a contracting trend compared with PMPC adsorption. To better understand the proposed mechanism, Figure 5 illustrates the schematic mechanism of PMPC and PCBMA adsorption behaviors on electrified ITO surfaces. As shown in Figure 4a, the PC moieties consist of a positively charged choline group exposed toward the surrounding aqueous medium and a negatively charged phosphate group linked to the main chain of PMPC. Therefore, PMPC polymer chains were illustrated as a cylinder structure with a neutral main chain surrounded by a negatively charged inner layer and a positively charged outer layer. When a positive surface potential is applied, PMPC will be repulsed due to its positively charged outer layer, and the dipole moment of PMPC acts against the electric field. The repulsive force reduces the attachment of PMPC on the ITO surface, resulting in a loose conformation of the PMPC thin layer. Conversely, applying a negative surface potential will attract the PMPC. The attractive force enhanced the attachment of PMPC to the ITO surface, resulting in a dense conformation of the PMPC thin layer. In contrast to the PMPC structure, CB moieties exposed the negatively charged carboxylate toward the surrounding aqueous medium and a positively charged choline group linked to the main chain of PCBMA. The opposite direction of dipole moments results in an opposite adsorption behavior compared to the PMPC illustrated in Figure 4b. Compared to the slight amount of adsorption and the little dependency on the surface potentials of zwitterionic MPC and CBMA monomers, PMPC and PCBMA exhibited distinct adsorption and followed the interaction between the charged surfaces and the charged polymer chains, originating from their direction of dipole moments on zwitterionic moieties. We attributed these different adsorption behaviors to the ability to rotate the dipole moments freely in the aqueous solutions, which polyzwitterions lack.

In the presence of 100 mM LiClO_4_, the |Δ*D*/Δ*f*| for PMPC adsorption in solutions is similar to that in DI water, although the adsorption amount of PMPC is reduced. The results show that the adsorbed PMPC thin layers present similar viscoelastic properties in the presence of 100 mM LiClO_4_, which indicates that the PMPC conformation is less dependent on ionic strength. However, in the presence of 100 mM LiClO_4_, the |Δ*D*/Δ*f*| values of PCBMA thin layers are approximately the same for all applied surface potentials, suggesting that the conformations of PCBMA layers remained similar regardless of their surface potential. We are currently not very clear about this mechanism, but we speculate that it may be due to the low adsorption of PCBMA in 100 mM LiClO_4_, with almost all of the polymers adhering to the ITO surface. Therefore, significant differences cannot be observed from |Δ*D*/Δ*f*|. This is also why the |Δ*D*/Δ*f*| values are particularly low. On the other hand, the |Δ*D*/Δ*f*| of PMPC shows a significantly weaker dependence on the applied potentials compared to that of PCBMA. According to prior research,^36,37^ it is known that the chain conformation PCBMA is more influenced by ionic strength compared to PMPC. PMPC displays robust interactions between PC groups and water molecules across both low- and high-ionic-strength solutions, with relatively weak intrachain interactions resulting in chain expansion. Conversely, PCBMA exhibits stronger intrachain interactions that are readily shielded by ions only at high ionic strength, facilitating polymer chain expansion. These results also indicate that the PCBMA conformation strongly depends on the ionic strength.

## Conclusions

This study demonstrated the adsorption behavior of two polyzwitterions, namely, PMPC and PCBMA, by applying an external surface potential using an EQCM-D. The PC moieties carry positively charged groups exposed outward to the surroundings and a negative one linked to the polymer main chains. In contrast, the CB moiety exhibited its charged groups in the opposite direction. The presence of these dipole moments in zwitterionic moieties provides a potential approach to electrochemically controlling the adsorption of these polyzwitterions based on the attraction or repulsion following the external electrical fields. The adsorption behavior of their monomers was examined first, and the effect of ionic strength on the electrified surfaces was evaluated by adding 100 mM LiClO_4_ to the aqueous solutions. The external surface potentials applied on the ITO surfaces affected the adsorption behavior of polyzwitterions. Based on the results of EQCM-D measurements, we shed light on understanding the relationship between polyzwitterion adsorption and electrified surfaces. In the future, we anticipate delving into the interactions among different zwitterionic polymers and electrified surfaces. Furthermore, we are eager to employ simulations for a more insightful and comprehensive quantitative analysis of this phenomenon.

## Supporting Information Available

Synthesis details and GPC results of PMPC and PCBMA molecular weight; ATR-FTIR spectrum of polyzwitterions; EQCM-D measurement on biased Au surfaces; EQCM-D measurement on biased Au surfaces; and Δ*f* of zwitterionic monomers (PDF)

## Abbreviations

PC: phosphorylcholine
SB: sulfobetaine
CB: carboxybetaine (CB)
MPC: 2-(methacryloyloxy)ethyl phosphorylcholine
PMPC: poly(2-methacryloyloxyethyl phosphorylcholine)
PCBMA: poly(carboxybetaine methacrylate)
ITO: indium–tin-oxide
LiClO_4_: lithium perchlorate
EQCM-D: electrochemical quartz crystal microbalance and dissipation

## References

[ref1] Li Q.; Wen C.; Yang J.; Zhou X.; Zhu Y.; Zheng J.; Cheng G.; Bai J.; Xu T.; Ji J.; et al. Zwitterionic Biomaterials. Chem. Rev. 2022, 122 (23), 17073–17154. 10.1021/acs.chemrev.2c00344. 36201481

[ref2] Erfani A.; Seaberg J.; Aichele C. P.; Ramsey J. D. Interactions between Biomolecules and Zwitterionic Moieties: A Review. Biomacromolecules 2020, 21 (7), 2557–2573. 10.1021/acs.biomac.0c00497. 32479065

[ref3] Schlenoff J. B. Zwitteration: Coating Surfaces with Zwitterionic Functionality to Reduce Nonspecific Adsorption. Langmuir 2014, 30 (32), 9625–9636. 10.1021/la500057j. 24754399 PMC4140545

[ref4] Mukai M.; Cheng C.-H.; Ma W.; Chin M.; Lin C.-H.; Luo S.-C.; Takahara A. Synthesis of a conductive polymer thin film having a choline phosphate side group and its bioadhesive properties. Chem. Commun. 2020, 56 (18), 2691–2694. 10.1039/C9CC09949B. 10.1039/C9CC09949B. 32051985

[ref5] Laschewsky A.; Rosenhahn A. Molecular Design of Zwitterionic Polymer Interfaces: Searching for the Difference. Langmuir 2019, 35 (5), 1056–1071. 10.1021/acs.langmuir.8b01789. 30048142

[ref6] Brown M. U.; Seong H.-G.; Russell T. P.; Emrick T. Zwitterionic Sulfonium Sulfonate Polymers: Impacts of Substituents and Inverted Dipole. Macromolecules 2023, 56 (3), 1105–1110. 10.1021/acs.macromol.2c02359.

[ref7] Lin C.-H.; Luo S.-C. Zwitterionic Conducting Polymers: From Molecular Design, Surface Modification, and Interfacial Phenomenon to Biomedical Applications. Langmuir 2022, 38 (24), 7383–7399. 10.1021/acs.langmuir.2c00448. 35675211

[ref8] Baggerman J.; Smulders M. M. J.; Zuilhof H. Romantic Surfaces: A Systematic Overview of Stable, Biospecific, and Antifouling Zwitterionic Surfaces. Langmuir 2019, 35 (5), 1072–1084. 10.1021/acs.langmuir.8b03360. 30620199 PMC6365910

[ref9] Wang W.; Lu Y.; Xie J.; Zhu H.; Cao Z. A zwitterionic macro-crosslinker for durable non-fouling coatings. Chem. Commun. 2016, 52 (25), 4671–4674. 10.1039/C6CC00109B 10.1039/C6CC00109B. 26952839

[ref10] Lowe A. B.; McCormick C. L. Synthesis and Solution Properties of Zwitterionic Polymers. Chem. Rev. 2002, 102 (11), 4177–4190. 10.1021/cr020371t. 12428987

[ref11] Delgado J. D.; Schlenoff J. B. Static and Dynamic Solution Behavior of a Polyzwitterion Using a Hofmeister Salt Series. Macromolecules 2017, 50 (11), 4454–4464. 10.1021/acs.macromol.7b00525.

[ref12] Kikuchi M.; Terayama Y.; Ishikawa T.; Hoshino T.; Kobayashi M.; Ohta N.; Jinnai H.; Takahara A. Salt Dependence of the Chain Stiffness and Excluded-Volume Strength for the Polymethacrylate-Type Sulfopropylbetaine in Aqueous NaCl Solutions. Macromolecules 2015, 48 (19), 7194–7204. 10.1021/acs.macromol.5b01116.

[ref13] Wang T.; Wang X.; Long Y.; Liu G.; Zhang G. Ion-Specific Conformational Behavior of Polyzwitterionic Brushes: Exploiting It for Protein Adsorption/Desorption Control. Langmuir 2013, 29 (22), 6588–6596. 10.1021/la401069y. 23659322

[ref14] Krishnamoorthy M.; Hakobyan S.; Ramstedt M.; Gautrot J. E. Surface-Initiated Polymer Brushes in the Biomedical Field: Applications in Membrane Science, Biosensing, Cell Culture, Regenerative Medicine and Antibacterial Coatings. Chem. Rev. 2014, 114 (21), 10976–11026. 10.1021/cr500252u. 25353708

[ref15] Muthukumar M. 50th Anniversary Perspective: A Perspective on Polyelectrolyte Solutions. Macromolecules 2017, 50 (24), 9528–9560. 10.1021/acs.macromol.7b01929. 29296029 PMC5746850

[ref16] Senechal V.; Rodriguez-Hernandez J.; Drummond C. Electroresponsive Weak Polyelectrolyte Brushes. Macromolecules 2022, 55 (7), 2636–2648. 10.1021/acs.macromol.1c02377.

[ref17] Pial T. H.; Prajapati M.; Chava B. S.; Sachar H. S.; Das S. Charge-Density-Specific Response of Grafted Polyelectrolytes to Electric Fields: Bending or Tilting?. Macromolecules 2022, 55 (7), 2413–2423. 10.1021/acs.macromol.2c00237.

[ref18] Blackman L. D.; Gunatillake P. A.; Cass P.; Locock K. E. S. An introduction to zwitterionic polymer behavior and applications in solution and at surfaces. Chem. Soc. Rev. 2019, 48 (3), 757–770. 10.1039/C8CS00508G 10.1039/C8CS00508G. 30548039

[ref19] Zurick K. M.; Bernards M. Recent biomedical advances with polyampholyte polymers. J. Appl. Polym. Sci. 2014, 131 (6), 40069 10.1002/app.40069.

[ref20] Kudaibergenov S. E.; Ciferri A. Natural and Synthetic Polyampholytes, 2. Macromol. Rapid Commun. 2007, 28 (20), 1969–1986. 10.1002/marc.200700197.

[ref21] Kamiyama Y.; Israelachvili J. Effect of pH and salt on the adsorption and interactions of an amphoteric polyelectrolyte. Macromolecules 1992, 25 (19), 5081–5088. 10.1021/ma00045a039.

[ref22] Khan M. O.; Åkesson T.; Jönsson B. Adsorption of Polyampholytes to Charged Surfaces. Macromolecules 2001, 34 (12), 4216–4221. 10.1021/ma001437u.

[ref23] Chen Y.; Luo S. C. Synergistic Effects of Ions and Surface Potentials on Antifouling Poly(3,4-ethylenedioxythiophene): Comparison of Oligo(Ethylene Glycol) and Phosphorylcholine. Langmuir 2019, 35 (5), 1199–1210. 10.1021/acs.langmuir.8b02122. 30089366

[ref24] Lin C. H.; Luo S. C. Combination of AFM and Electrochemical QCM-D for Probing Zwitterionic Polymer Brushes in Water: Visualization of Ionic Strength and Surface Potential Effects. Langmuir 2021, 37 (42), 12476–12486. 10.1021/acs.langmuir.1c02230. 34648298

[ref25] Wu J. G.; Wei S. C.; Luo S. C. In Situ Probing Unusual Protein Adsorption Behavior on Electrified Zwitterionic Conducting Polymers. Adv. Mater. Interfaces 2020, 7 (15), 2000470 10.1002/admi.202000470.

[ref26] Lemineur J.-F.; Ciocci P.; Noël J.-M.; Ge H.; Combellas C.; Kanoufi F. Imaging and Quantifying the Formation of Single Nanobubbles at Single Platinum Nanoparticles during the Hydrogen Evolution Reaction. ACS Nano 2021, 15 (2), 2643–2653. 10.1021/acsnano.0c07674. 33523639

[ref27] Lai B. C.; Wu J. G.; Luo S. C. Revisiting Background Signals and the Electrochemical Windows of Au, Pt, and GC Electrodes in Biological Buffers. ACS Appl. Energy Mater. 2019, 2 (9), 6808–6816. 10.1021/acsaem.9b01249.

[ref28] Chockalingam M.; Magenau A.; Parker S. G.; Parviz M.; Vivekchand S. R. C.; Gaus K.; Gooding J. J. Biointerfaces on Indium-Tin Oxide Prepared from Organophosphonic Acid Self-Assembled Monolayers. Langmuir 2014, 30 (28), 8509–8515. 10.1021/la501774b. 24960524

[ref29] Gaddam P.; Ducker W. Electrostatic Screening Length in Concentrated Salt Solutions. Langmuir 2019, 35 (17), 5719–5727. 10.1021/acs.langmuir.9b00375. 30945875

[ref30] Berg J. C. An Introduction to Interfaces & Colloids: The Bridge to Nanoscience; World Scientific, 2010.

[ref31] Israelachvili J. N. Intermolecular and Surface Forces; Elsevier Science, 2015.

[ref32] Israelachvili J. N.; Adams G. E. Measurement of forces between two mica surfaces in aqueous electrolyte solutions in the range 0–100 nm. J. Chem. Soc., Faraday Trans. 1 1978, 74 (0), 975–1001. 10.1039/F19787400975 10.1039/f19787400975.

[ref33] Molino P. J.; Higgins M. J.; Innis P. C.; Kapsa R. M. I.; Wallace G. G. Fibronectin and Bovine Serum Albumin Adsorption and Conformational Dynamics on Inherently Conducting Polymers: A QCM-D Study. Langmuir 2012, 28 (22), 8433–8445. 10.1021/la300692y. 22551342

[ref34] Dolatshahi-Pirouz A.; Jensen T.; Foss M.; Chevallier J.; Besenbacher F. Enhanced Surface Activation of Fibronectin upon Adsorption on Hydroxyapatite. Langmuir 2009, 25 (5), 2971–2978. 10.1021/la803142u. 19437707

[ref35] Zhang S. L.; Bai H. H.; Yang P. H. Real-time monitoring of mechanical changes during dynamic adhesion of erythrocytes to endothelial cells by QCM-D. Chem. Commun. 2015, 51 (57), 11449–11451. 10.1039/C5CC03264D. 26087999

[ref36] Higaki Y.; Kobayashi M.; Takahara A. Hydration State Variation of Polyzwitterion Brushes through Interplay with Ions. Langmuir 2020, 36 (31), 9015–9024. 10.1021/acs.langmuir.0c01672. 32677837

[ref37] Zhang Z.; Vaisocherová H.; Cheng G.; Yang W.; Xue H.; Jiang S. Y. Nonfouling Behavior of Polycarboxybetaine-Grafted Surfaces: Structural and Environmental Effects. Biomacromolecules 2008, 9 (10), 2686–2692. 10.1021/bm800407r. 18785772

