# Supplementary material for: Electrified Interactions of Polyzwitterions with Charged Surfaces: Role of Dipole Orientation and Surface Potentials
Source: Langmuir. 2024 Mar 27;40(14):7653–60. doi: 10.1021/acs.langmuir.4c00343 (PMC11008249; doi:10.1021/acs.langmuir.4c00343)
Supplement: Supplementary file 1 — la4c00343_si_001.pdf [file la4c00343_si_001.pdf]

Supporting Information

Electrified Interactions of Polyzwitterions with Charged Surfaces: Role of Dipole Orientation and Surface Potentials

Chia-Hsuan Lin,[†] *Jhih-Guang Wu,*[†] *Hsun-Hao Lin,*[†] *and Shyh-Chyang Luo**[†,‡]

[†]Department of Materials Science and Engineering, National Taiwan University, No. 1, Sec. 4, Roosevelt Road, Taipei 10617, Taiwan.

[‡]Institute of Biomedical Engineering and Nanomedicine, National Health Research Institutes (NHRI), Miaoli County, 35053 Taiwan

*Email: shyhchyang@ntu.edu.tw

Table of Contents

Experimental Section

Table S1: The mole and equivalent ratio for ATRP to obtain polyzwitterions.

Figure S1: Molecular weights of polyzwitterions measured by GPC.

Figure S2: ATR-FTIR spectrum of polyzwitterions.

Figure S3: Unstable signals during EQCM-D measurement on biased Au surfaces.

Figure S4: The real-time Δf during the adsorption process of zwitterionic monomers with a concentration of 10 mg/ml in DI water on the ITO surface.

Table S1. The mole and equivalent ratio for ATRP to obtain polyzwitterions.

	Monomer (mmol)	EBIB (mmol)	CuBr (mmol)	Bpy (mmol)
PMPC	8.4	0.042	0.042	0.084
Equivalent ratio	200	1	1	2
PCBMA	5	0.025	0.025	0.05
Equivalent ratio	200	1	1	2

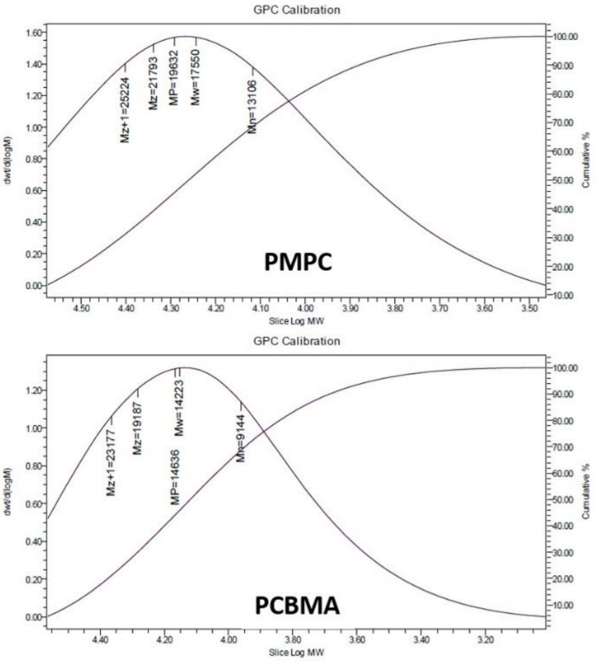

Table of molecular weight data

	PMPC	PCBMA
M_w	17550	14223
M_n	13106	9144
DP (M_n)	44.38	39.93
M_w/M_n	1.34	1.55

Figure S1. GPC results of PMPC, and PCBMA prepared by ATRP methods. The table summarised the number of repeat units and polydispersity index of these polymers.

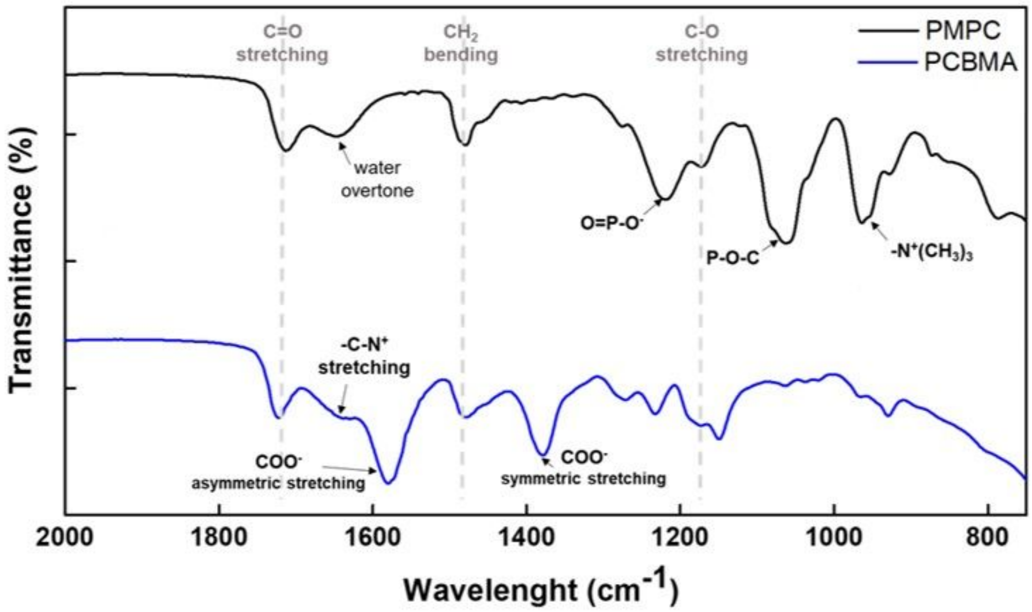

Figure S2. ATR-FTIR spectrum of PMPC, and PCBMA polymers.

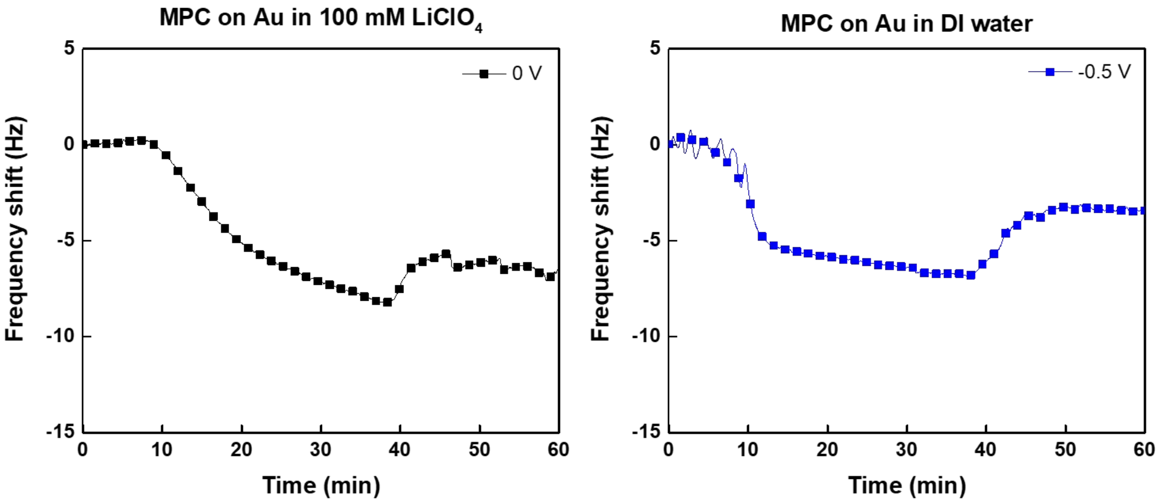

Figure S3. The real-time Δf on the biased Au surfaces during the EQCM-D measurements with unstable signals: the continuous steps (left) and the strong noises (right).

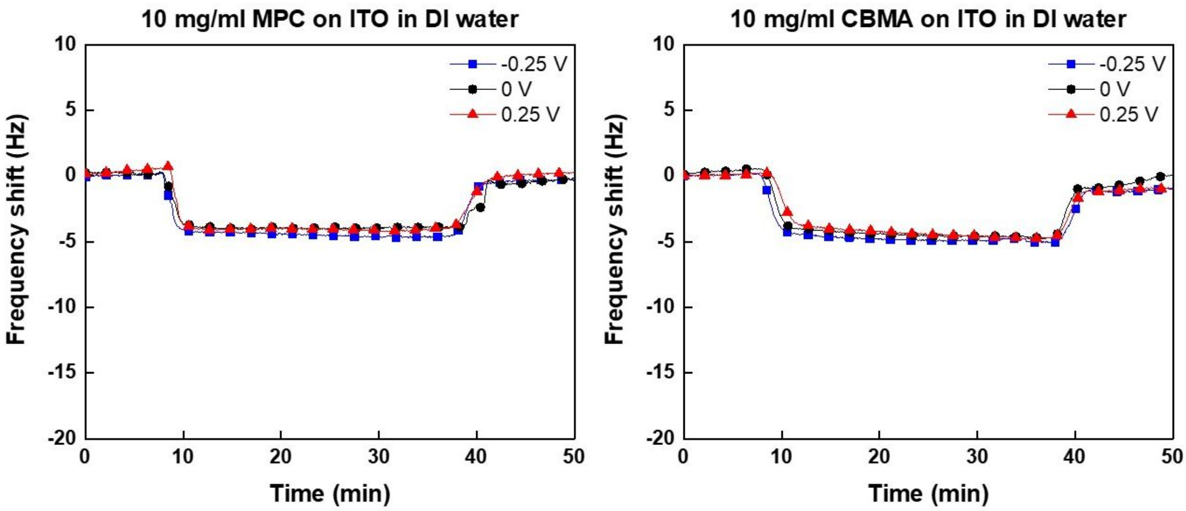

Figure S4. The real-time Δf during the adsorption process of zwitterionic monomers with a concentration of 10 mg/ml in DI water on the ITO surface with an external potential of −0.25, 0, and 0.25 V.
